# Mass Spectrometric Comparison of HPV-Positive and HPV-Negative Oropharyngeal Cancer

**DOI:** 10.3390/cancers12061531

**Published:** 2020-06-11

**Authors:** Marcus Wurlitzer, Nikolaus Möckelmann, Malte Kriegs, Maren Vens, Maryam Omidi, Konstantin Hoffer, Clara von Bargen, Christina Möller-Koop, Melanie Witt, Conrad Droste, Agnes Oetting, Hannes Petersen, Chia-Jung Busch, Adrian Münscher, Hartmut Schlüter, Till Sebastian Clauditz, Thorsten Rieckmann

**Affiliations:** 1 Mass Spectrometric Proteomics Group, Institute of Clinical Chemistry and Laboratory Medicine, University Medical Center Hamburg Eppendorf, 20246 Hamburg, Germany; m.wurlitzer@uke.de (M.W.); m.omidi@uke.de (M.O.); hschluet@uke.de (H.S.); 2 Department of Otorhinolaryngology, University Medical Center Hamburg Eppendorf, 20246 Hamburg, Germany; n.moeckelmann@uke.de (N.M.); a.oetting@uke.de (A.O.); petersen@hno-europahaus.de (H.P.); cjbusch@uke.de (C.-J.B.); muenscher.hno@marienkrankenhaus.org (A.M.); 3 Department of Radiotherapy and Radiation Oncology, University Medical Center Hamburg Eppendorf, 20246 Hamburg, Germany; m.kriegs@uke.de (M.K.); k.hoffer@uke.de (K.H.); 4 Department of Medical Biometry and Epidemiology, University Medical Center Hamburg Eppendorf, 20246 Hamburg, Germany; m.vens@uni-luebeck.de; 5 Institute of Medical Biometry and Statistics, University of Lübeck, 23562 Lübeck, Germany; 6 Institute of Pathology, University Medical Center Hamburg Eppendorf, 20246 Hamburg, Germany; c.von-bargen@uke.de (C.v.B.); c.moeller-koop@uke.de (C.M.-K.); me.witt@uke.de (M.W.); t.clauditz@uke.de (T.S.C.); 7 University Cancer Center Hamburg (UCCH), University Medical Center Hamburg Eppendorf, 20246 Hamburg, Germany; c.droste@uke.de; 8 Department of Otorhinolaryngology, Marienkrankenhaus, 22087 Hamburg, Germany

**Keywords:** head and neck cancer, oropharynx, HPV, mass spectrometry

## Abstract

Squamous cell carcinoma of the head and neck (HNSCC) consist of two distinct biological entities. While the numbers of classical, tobacco-induced HNSCC are declining, tumors caused by human papillomavirus (HPV) infection are increasing in many countries. HPV-positive HNSCC mostly arise in the oropharynx and are characterized by an enhanced sensitivity towards radiotherapy and a favorable prognosis. To identify molecular differences between both entities on the protein level, we conducted a mass spectrometric comparison of eight HPV-positive and nine HPV-negative oropharyngeal tumors (OPSCC). Overall, we identified 2051 proteins, of which 31 were found to be differentially expressed. Seventeen of these can be assorted to three functional groups, namely DNA replication, nuclear architecture and cytoskeleton regulation, with the differences in the last group potentially reflecting an enhanced migratory and invasive capacity. Furthermore, a number of identified proteins have been described to directly impact on DNA double-strand break repair or radiation sensitivity (e.g., SLC3A2, cortactin, RBBP4, Numa1), offering explanations for the differential prognosis. The unequal expression of three proteins (SLC3A2, MCM2 and lamin B1) was confirmed by immunohistochemical staining using a tissue microarray containing 205 OPSCC samples. The expression levels of SLC3A2 and lamin B1 were found be of prognostic relevance in patients with HPV-positive and HPV-negative OPSCC, respectively.

## 1. Introduction

In recent decades, there has been an increase in the incidence of head and neck squamous cell carcinomas (HNSCC) with location in the oropharynx (OPSCC), whilst tumors arising from the larynx and hypopharynx have been declining. Sustained alcohol and tobacco consumption no longer represent the main risk factors for OPSCC in many countries as smoking cessation programs were implemented [1]. The rising numbers of OPSCC can be largely ascribed to an epidemic spread of human papillomavirus (HPV)-associated tumors mostly located at the tonsils and the base of the tongue. In the US, the incidence of HPV-positive OPSCC has increased by 225% between 1988 and 2004, and HPV-negative OPSCC declined by 50%. Similar trends are being observed in many other developed countries [2,3,4,5]. HPV-negative and HPV-positive HNSCC represent biologically and clinically distinct entities. The tumorigenesis of HPV-positive cancer is largely determined by the activity of the viral oncoproteins E6 and E7. Amongst many other functions, E6 and E7 inhibit and degrade the tumor suppressors p53 and the retinoblastoma-associated protein (pRB), respectively. pRB degradation results in the activation of the transcription factors of the E2F-family, which drive S-phase entry. E2Fs also induce the expression of the CDK-inhibitor p16, which, in a negative feedback loop, restricts E2F-activity by reinstalling the active hypophosphorylated form of pRB [6,7]. Since in HPV-positive tumors, this regulatory loop is broken by the degradation of pRB through E7, these tumors express and tolerate large amounts of p16. In HPV-negative OPSCC, p16 is usually suppressed and therefore, p16 is commonly used as a surrogate marker for HPV-positivity in the clinics. Patients with HPV-positive OPSCC show a clearly favorable prognosis despite frequent presentation with lymph node metastasis. The higher survival rates are attributed to an enhanced sensitivity towards therapy and are independent of the choice of primary treatment modality [8,9]. From data with sole radiotherapy treatment, an enhanced sensitivity of HPV-positive OPSCC toward radiation is clearly established [10,11] and in locally advanced disease, radiotherapy is virtually always included in current multimodal regimes. Proposed mechanisms for the higher (radio) sensitivity are enhanced immune responses [12,13,14] and a higher intrinsic radiation sensitivity of HPV-positive tumor cells, mostly described to be caused by a reduced ability to repair radiation induced DNA double-strand breaks [13,15,16,17].

To identify biologically relevant differences in protein expression between HPV-positive and HPV-negative OPSCC in an open, unbiased experimental approach, we conducted a mass spectrometric comparison of the proteomes of HPV-positive and HPV-negative OPSCC tissue samples.

## 2. Material and Methods

### 2.1. Patient Characteristics

Patients treated for HNSCC at the Department of Otorhinolaryngology of the University Medical Center Hamburg-Eppendorf between August 2011 and March 2013 were reviewed for primary site and HPV/p16 status within our clinical cancer database. p16 status had been determined by immunohistochemistry using a mouse anti-p16^INK4a^ antibody (clone G175-405; BD Biosciences, Heidelberg, Germany) and HPV status had been determined by genomic PCR using the MY09/11 primer set and subsequent sequencing of PCR products. Ten patients with HPV/p16-positive and 10 with HPV/p16-negative OPSCC with leftover formalin-fixed paraffin-embedded (FFPE) tumor tissue available from histopathological workup of the main tumor specimen were chosen for the mass spectrometric comparison. The tissue microarray (TMA) contains tumor samples from patients who had been diagnosed with a squamous cell carcinoma of the oropharynx and treated with curative intent at the University Medical Center Hamburg-Eppendorf between 1992 and 2013. The use of archived diagnostic leftover tissues and their analysis for research purposes as well as patient data analysis have been approved by local laws (HmbKHG,§12,1) and by the local ethics committee (Ethics commission Hamburg, WF-049/09). The whole study has been carried out in compliance with the Helsinki Declaration.

### 2.2. Sample Preparation and Processing

#### 2.2.1. Tissue Sectioning and Protein Extraction

The FFPE tissue blocks from HPV-positive and HPV-negative tumors were sliced with a microtome into sections of 6 µm thickness and transferred onto specimen slides. A total of 22 sections were cut from each tissue sample. The first and last specimens were stained with haematoxylin and eosin (HE) for microscopical assessment of the tumor. Areas of tumor growth were marked by a pathologist (T.S.C.) on both HE slides to provide guidance for the macrodissection of slides 2 to 21 in the next step.

The tissue slices were washed subsequently by xylene (ChemSolute, Th. Geyer, Renningen, Germany) twice, 100% ethanol (Merck, Darmstadt, Germany) twice, and once in 95%, 70% and 30% ethanol for 10 min for each washing step for deparaffinization. Then, tissues from tumor areas were transferred from the slides to reaction vials with a scalpel and incubated at 65 °C in antigen retrieval buffer (Retrievit TM2, Target Retrieval Solution 10× (BioGenex, Fremont, CA, USA)) for 4 h. Then, the tissues were washed twice with HPLC-grade water (LiChrosolv (Merck, Darmstadt, Germany) and centrifuged at 12,000 rpm for 5 min.

#### 2.2.2. Tryptic Digestion and Desalting

A quantity of 200 µL of 10 mM dithiothreitol (Sigma-Aldrich Chemie, (Aufkirchen, Germany)) dissolved in a 100 mM ammonium bicarbonate solution (NH_4_HCO_3_ (Carl Roth GmbH, Karlsruhe, Germany)) in HPLC-grade water were added to the reaction vials for reduction of cysteine residues, incubated for 10 min at 56 °C followed by centrifugation at 12,000 rpm for 5 min. Cysteine residues were alkylated with 300 mM iodoacetamide (Sigma-Aldrich Chemie) dissolved in 100 mM NH_4_HCO_3_. The samples were incubated in the dark at room temperature for 20 min, followed by centrifugation at 12,000 rpm for 5 min and removal of the supernatant. Finally, 200 µL sequencing grade modified trypsin (Promega, Madison, WI, USA) was dissolved to a final concentration of 0.1 µg/µL in HPLC-grade water and 1M ammonium bicarbonate of 100:4) was added to the tissue slices and incubated overnight at 37 °C. Finally, the samples were centrifuged in 12,000 rpm for 10 min and the supernatant was collected and transferred to new collection vials and dried with a vacuum concentrator.

The samples were desalted using Poros Oligo R3 reversed-phase packing material (Oligo R3 Bulk Medium (Applied Biosystems, Darmstadt, Germany) and a C18-EMPORE-DISC (Sigma-Aldrich, Steinheim, Germany). Single-use desalting columns were prepared by stamping out a 6 mm piece of the C18 disc and placing it into a gel loader tip, followed by washing with 100% acetonitrile (ACN, LiChrosolv, Merck). Then, 50 μL of Oligo R3 dissolved in 50% ACN were added on top of each column. The columns were conditioned by washing with 60 μL elution buffer (50% ACN in HPLC-grade water with 0.1% trifluoroacetic acid (TFA (Sigma-Aldrich)) and equilibrated with 60 μL wash buffer (0.1% TFA in HPLC-grade water). Then, the sample was dissolved in wash buffer and loaded onto the column, and the peptides were eluted with the elution buffer. The eluates were dried with a vacuum concentrator and stored frozen until mass spectrometric analysis was carried out.

#### 2.2.3. LC–MS/MS Analysis

Dried samples were dissolved in a mixture of 2 µL 50% ACN and 18 µL 0.2% formic acid (FA, Merck) in HPLC-grade water, then diluted 1:10 in 0.2% FA and centrifuged for 10 min at 15,000 rpm at 4 °C. The tryptic peptides were subjected to a reversed-phase nano-UPLC (Dionex UltiMate 3000 RSLCnano (Thermo Fisher Scientific, Bremen, Germany)) coupled via electrospray ionization (ESI) to an Orbitrap mass spectrometer (Orbitrap Fusion (Thermo Fisher Scientific)). Samples were injected onto a trapping column (Acclaim PepMap μ-precolumn, C18, 300 μm × 5 mm, 5 μm, 100 Ǻ (Thermo Scientific, Dreieich; Germany)) by an autosampler at 98% buffer A (0.1% FA in HPLC-water) and 2% buffer B (0.1% FA in ACN) and washed with 2% buffer B. The peptides were separated on a separation column (Acclaim PepMap 100, C18, 75 μm × 250 mm, 2 μm, 100 Ǻ (Thermo Scientific)) by a gradient of 2% to 30% B in 90 min at a flow rate of 200 nL/min, followed by a gradient to 70% B in 10 min, another gradient to 90% B in 2 min and holding 90% B for 3 min before the column was equilibrated at 2% B for 15 min for the next run.

The peptides eluting from the column were ionized with electrospray ionization in positive mode. The mass spectrometer was operating in data-dependent acquisition top-speed mode with the following parameters: MS1 scan range of 400–1500 Th, MS1 resolution of 120,000, AGC target of 4e5, maximum injection time of 50 ms. Ions with charge states 2–6 and a minimum intensity of 1e5 were selected for fragmentation, with a dynamic exclusion for 30 s for previously fragmented ions. Selected precursor ions were isolated with a width of 1.5 Th and fragmented at a HCD collision energy of 35%. MS2 spectra were acquired with the ion trap in rapid mode with a maximum injection time of 50 ms and an AGC target of 1e4.

### 2.3. Peptide Identification and Quantitative Proteomics

Raw LC–MS data were processed with MaxQuant (Version 1.5.3.30, Max-Planck Institute of Biochemistry, Munich, Germany) [18]. Peptide identification was performed with the Andromeda search engine against the human SwissProt database (www.uniprot.org, 20,161 entries) and the internal contaminant database. The search parameters were set as follows: the precursor mass tolerance was set to 8 ppm, the fragment mass tolerance was set to 0.5 Da, and two missed cleavages were allowed for peptide identification; an FDR of 1% was given on the peptide and the protein level. Carbamidomethylation of cysteine residues was set as a fixed modification. Oxidation of methionine residues and acetylation of the protein N-terminus were set as variable modifications (five at most for each peptide). The MaxLFQ algorithm [19] was used for protein quantification with a minimum ratio count of 1.

To compare the protein intensities between the HPV+ and HPV− groups, the quantitative protein data was loaded into Perseus 1.5.8.5 [20]. First, label-free quantification (LFQ) intensities were logarithmized to base 10. Missing values were imputed from a down-shifted normal distribution. The HPV+ and HPV− groups were compared with a two-sided t-test. Only proteins with at most two missing values in at least one group were considered.

### 2.4. Random Forest Analysis

R and the Bioconductor environment [21] were used to build random forests in order to separate the HPV-positive and HPV-negative group. In total, 100 runs were performed. The Boruta package was used to decide whether a protein was important for the separation of groups. If importance was confirmed in one run, the protein was scored 2, if importance was tentative, it was scored 1 and if the importance was rejected by the Boruta algorithm, the protein was scored 0. Subsequently, the Boruta sum score was built for each protein. Proteins reaching a score of ≥10 were rated as differentially expressed.

### 2.5. Analyses of Identified Proteins

The list of differentially expressed proteins was uploaded to the STRING database [22] to visualize predicted interactions. A heat map of logarithmic protein intensities of the differential proteins was created in Perseus, depicting the relative protein abundance compared to the median, with row clustering enabled to group proteins of similar expression patterns. Depiction of individual protein intensities, student’s *t*-test and regression analyses were performed using GraphPad Prism 6. Graphs depict mean values and standard error (SE) unless stated otherwise.

### 2.6. Tissue Microarray (TMA) Analysis

TMA construction was described previously [23,24]. For immunohistochemistry (IHC) analyses, freshly cut 3 μm thick TMA sections were analyzed on the same day in a single experiment. Proteins of interest were stained using specific antibodies (rabbit anti-NUP210 (Sigma, Taufkirchen, Germany, HPA066888)), mouse anti-MCM2 (Thermo Fisher Scientific, Bremen, Germany, clone 1E7, 1:450), rabbit anti-SLC3A2 (Fitzgerald, Acton, MA, USA, 70R-12779, 1:50), rabbit anti-LRPPRC (Abcam, Cambridge, UK, ab97505, 1:450), rabbit anti-Lamin B1 (Atlas antibodies, Bromma, Sweden, HPA050524, 1:350) after peroxidase blocking with H2O2 (DAKO S2023 (Agilent, Santa Clara, CA, USA)) for 10 min. High-temperature pretreatment of slides was done in an autoclave with citrate buffer, pH 7.8 or pH 9 for 5 min. The Envision system (DAKO K5007 (Agilent)) was used to visualize the immunostaining. Individual spots were scored for staining intensity (0, 1, 2 or 3 referring to negative, weak, moderate or strong staining) and for the fraction of tumor cells in the highest category. To be assorted to the intensity categories 2 and 3, a minimum threshold of 30% tumor cells had to be reached.

### 2.7. Analysis of Patient Survival

R and the Bioconductor environment [21] were used for data processing, analysis and evaluation. Survival analyses were performed according to the Kaplan–Meier method and the Log-rank test using the R packages “survival” and “survminer” [25,26]. For correlation analysis, we used “reshape2” for data processing and “corrplot” for data analysis and visualization [27,28].

## 3. Results

**Figure 1:**
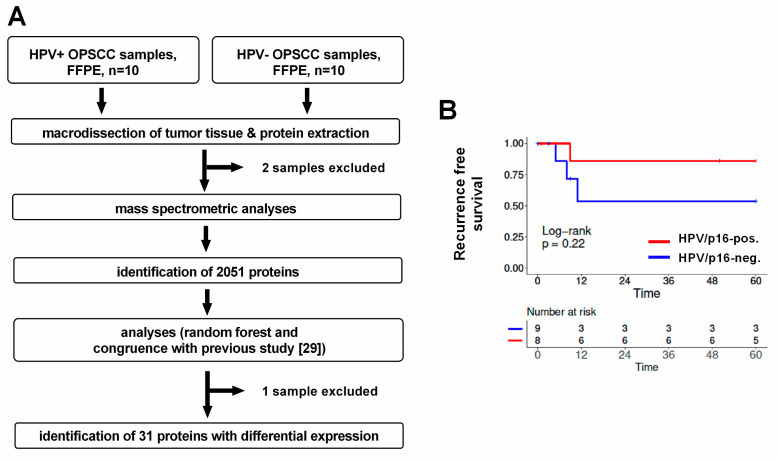
Experimental workflow and patient survival. (**A**) 10 HPV-positive and 10 HPV-negative OPSCC were initially chosen for the analysis. Two extracts were excluded from the mass spectrometric analysis because of insufficient amounts of protein. A single HPV-negative sample was further excluded from the random forest analysis because its expression pattern severely impaired group separation. For reasons of comparability, the sample was also excluded from protein identification based on congruence with a comparable previous study (through *p*-value and fold change) although a control analysis demonstrated only a marginal influence of the sample in this approach. (**B**) Recurrence-free survival of the 17 patients whose tumors were included in the final analyses.

To characterize proteome differences, we conducted a mass spectrometric comparison of primary HPV-positive and HPV-negative OPSCC. The experimental workflow is outlined in Figure 1A. The final analyses contained eight HPV-positive and nine HPV-negative tumors. To avoid protein expression differences governed by HPV-independent factors, such as degree of hypoxia, we had chosen cohorts with similar tumor size (T-stage) for the analysis. In line with their typical clinical appearance, all HPV-positive tumors were characterized by some degree of lymph node metastasis but favorable patient survival (Table 1, Figure 1B). The HPV-positive and HPV-negative cohorts were not found to be statistically different regarding age, smoking status, T-, N-, or UICC-status. Liquid chromatography–tandem mass spectrometry (LC–MS/MS) analysis of trypsin-digested, formalin-fixed tissue samples identified a total of 2051 proteins with at least one unique peptide.

The complete list of proteins including their respective intensity values in the individual tumors is presented in Table S1. Two methods were used to detect differential expression between the two groups. Primarily, we applied a random forest machine learning approach to identify specific proteins that are able to distinguish between the two groups. Additionally, proteins were defined as unequally expressed when the intensity values were significantly different between the two groups in a two-sided t test (*p* < 0.05; non-adjusted), the group means were at least different by a factor of 2 and corresponding observations had been made in a previous, comparable mass spectrometric study [29].

**Figure 2:**
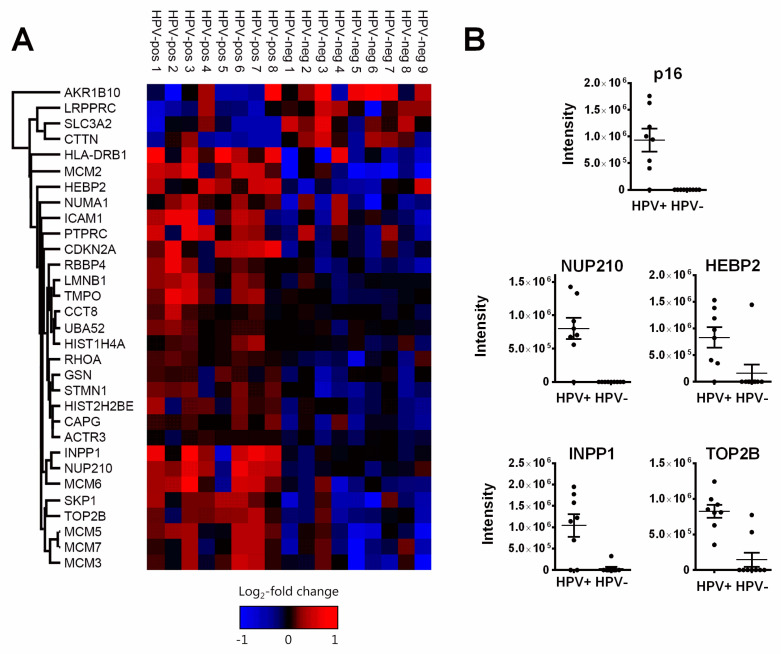
Proteins differentially expressed in HPV-positive and HPV-negative OPSCC. (**A**) Heat map depicting the differentially expressed proteins. Only four proteins were found to be expressed at a higher level in HPV-negative OPSCC. (**B**) Expression levels of proteins (almost) exclusively detected in HPV-positive OPSCC as assessed by LC–MS/MS intensity values. Note that for (**A**) and in the statistical analyses, random low-intensity values were assigned to proteins not detected in the LC–MS/MS measurement to avoid false-positive significance.

The random forest analysis identified a total of 24 proteins whose differential expression allowed separation of the HPV-positive and HPV-negative group in at least 5 of the 100 runs performed. A total of 15 proteins were concordantly identified through *p*-value and fold change in our and a similar study by Sepiashvili et al. [29]. With seven of these not identified in the random forest analyses, we obtained a total of 31 differentially expressed proteins. Surprisingly, only four of these (SLC3A2, LRPPRC, Cortactin, AKR1B10) were expressed at a higher level in HPV-negative tumors, while 27 were expressed at a higher level in HPV-positive (Table 2, Figure 2A, Figure S1).

A prerequisite for the inclusion of clinical samples in our study was positivity, respectively negativity for immunohistochemical p16 staining in the HPV-positive or -negative cohort. In line with this, p16 (product of the *CDKN2A* gene) was exclusively detected in HPV-positive tumors. It was classified as differentially expressed by both definitions and among a group of six proteins identified in every run of the random forest analysis. Very similar patterns of (almost) exclusive identification in HPV-positive tumors were observed for four other proteins: Nuclear pore membrane glycoprotein 210 (NUP210), heme-binding protein 2 (HEBP2 or SOUL), inositol polyphosphate 1-phosphatase (INPP1) and topoisomerase 2 beta (TOP2B) (Figure 2B). Due to their similar expression patterns to p16, these proteins may have the potential to serve as additional surrogate markers for HPV-induced tumors, enabling a more specific immunohistochemistry (IHC)-based discrimination in OPSCC and possibly also in non-OPSCC, where sole p16 staining is clearly insufficient [30].

### 3.1. Pathways and Functions of Identified Proteins

More than half of the 27 proteins upregulated in HPV-positive OPSCC could be assigned to one of three distinct functional groups: 1. DNA replication, 2. Nuclear architecture and 3. Regulation of the cytoskeleton (Figure S2).

#### 3.1.1. DNA Replication Factors (MCM2/3/5/6/7, RBBP4)

**Figure 3:**
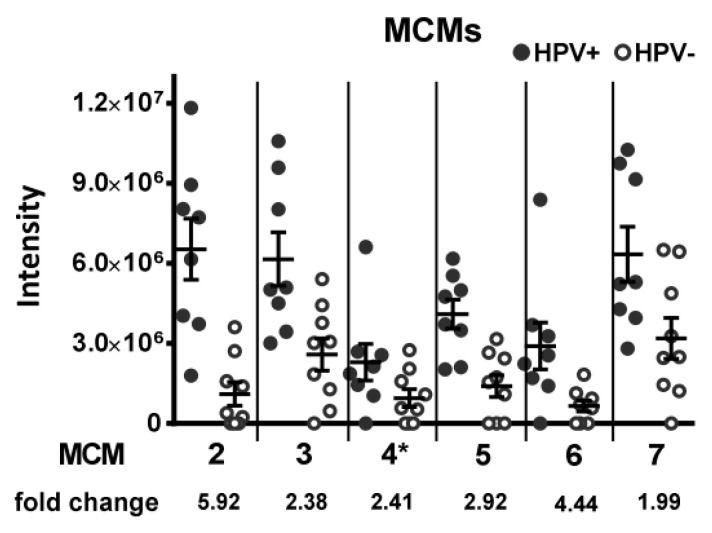
Expression of minichromosome maintenance proteins. All subunits of the MCM complex are, on average, expressed at a higher level in HPV-positive OPSCC, as assessed by LC–MS/MS intensity values. * not identified to be differentially expressed in our analyses.

This group includes five of the six minichromosome maintenance homolog proteins (MCM), which form the replicative helicase complex, a hexameric ring that separates the DNA double-strand preceding the replication fork. In fact, all detected MCM proteins showed higher expression levels in HPV-positive tumors (Figure 3).

The MCM complex is critical for replication initiation as well as replication fork progression [31]. A dissociation of the complex from the rest of the replication fork machinery is a hallmark of replication stress and exposes stretches of single-stranded DNA, which is at constant risk of nuclease digestion possibly contributing to genomic instability. The expression levels of the individual MCM subunits are interconnected and the downregulation of single subunits negatively influences the expression levels of others [32,33]. In line with this, our measurements demonstrate tight associations of the expression levels of various MCM proteins in the individual tumor samples, indicating that MCM expression follows an orchestrated pattern, also in the HPV+ tumors with enhanced expression (Figure S3). Also expressed at a higher level are the retinoblastoma-binding proteins 4/7 (RBBP4/7 or RbAp48/46), histone-binding proteins and part of the chromatin assembly factor complex 1 (CAF-1). CAF-1 is directly attached to the replication fork and deposits histone A3/A4 dimers onto newly replicated DNA, promoting rapid chromatin reassembly [34,35].

#### 3.1.2. Nuclear Architecture (Lamin B1, LAP2, NUP210, Numa1)

**Figure 4:**
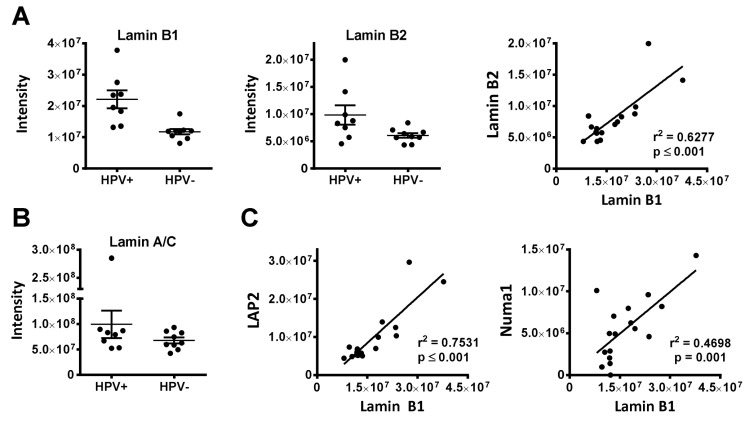
Nuclear envelope proteins. (**A**) Expression and association of the expression levels of B-type lamins as assessed by LC–MS/MS intensity values. (**B**) Similar expression of LMNA in HPV-positive and –negative OPSCC. (**C**) Significant associations of the expression levels of lamin B1 and LAP2 and lamin B1 and Numa1, respectively.

Four proteins upregulated in HPV+ OPSCC represent structural components of the nucleus and nuclear envelope. Nuclear mitotic apparatus protein 1 (Numa1) is one of the most abundant structural components of the interphase nucleus and has been suggested to be a major constituent of the proposed nuclear scaffold termed the nuclear matrix [36]. Nuclear Numa 1 serves diverse functions, e.g., in chromatin organization [37,38] and DNA repair [39,40]. Interestingly, it has been shown to directly interact with HPV oncoproteins [41,42]. Lamin B1 and lamina-associated polypeptide 2 (LAP2 or thymopoietin, TMPO) are components of the nuclear lamina and membrane, while NUP210 is a component of the nuclear pores, which enable traffic through the aforementioned structures. The lamins A, C, B1 and B2 build the core of the nuclear lamina, which is linked to the inner nuclear membrane and to chromatin through LEM-domain proteins, such as LAP2. The nuclear lamina and associated proteins provide structural support for the nucleus and serve a wide range of functions, including chromatin organization, regulation of replication, gene expression and DNA repair as well as telomere maintenance and signaling (reviewed in detail in [43]). We observed a higher expression of lamin B1 in HPV-positive OPSCC and a tight association between lamin B1 and B2, although the latter was not identified in our analyses (Figure 4A). The expression of lamins A/C (both derived from the *LMNA* gene through alternative splicing) was similar in both groups (Figure 4B). A subset of HPV-positive OPSCC may, therefore, possess a different composition of the nuclear lamina with a higher abundance of B-type lamins. LAP2 is expressed in different isoforms, partly interacting with lamin B1 and partly with the nucleoplasmic subfraction of lamins A/C. The expression of lamin B1 and LAP2 was recently reported to be simultaneously regulated (together with pRB) by the ubiquitin ligase RNF123 (RING finger protein 123) [44]. In line with this, we observe a significant association of their expression levels and, interestingly, the expression of lamin B1 and Numa1 were also significantly associated (Figure 4C).

NUP210 is one of only two transmembrane proteins of the nuclear pore complex but is not necessarily required for either assembly or function and is lacking in some cell types [45,46,47]. It was found to be overexpressed in a number of tumors, such as ovarian cancer, often together with lamin B1 and 2 [48]. It was reported to inhibit apoptosis in the context of muscle cell development and in peripheral CD4^+^ T cells [49,50]. Whether NUP210 may also serve an anti-apoptotic function in cancer cells is currently unknown.

#### 3.1.3. Regulators of the Cytoskeleton (APR3, CAPG, Gelsolin, Stathmin, Numa1, CCT8, RhoA/B/C)

**Figure 5:**
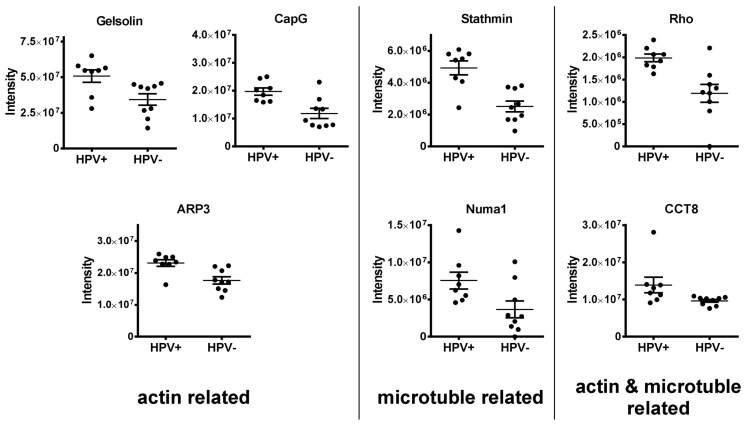
Cytoskeleton regulators. Depicted are the individual expression levels of cytoskeleton organizing and regulating proteins with differential expression in HPV-positive and HPV-negative OPSCC as assessed by LC–MS/MS intensity values.

This group comprises seven actin and microtubule organizing proteins (Figure 5). The cytoskeleton is composed of actin filaments, microtubules and intermediate filaments. It provides shape and stability, is required for migration, intracellular transport of vesicles and organelles, nuclear and cell division and influences cellular signaling [51]. Actin-related protein 3 (ARP3) is part of the ARP2/3 complex, which promotes the formation of branched actin networks [52], gelsolin is a versatile actin-regulating protein that can sever existing filaments, block their barbed ends against elongation but can also promote filament assembly [53]. The related protein CapG (macrophage-capping protein) also blocks filament ends but does not sever existing filaments [54]. Stathmin prevents the polymerization of tubulin and promotes the depolymerization of microtubules, while Numa1 is the major microtubule tethering factor at the mitotic spindle [36,55]. The Rho-GTPases are signaling transducers that critically regulate various factors and pathways involved in both actin filament and microtubule formation [56], whereas CCT8 (chaperonin containing T-complex polypeptide 1 subunit 8) as part of the TRiC chaperone complex assists the folding of actin and tubulin [57]. The enhanced expression of these factors points to enhanced cytoskeletal dynamics in HPV-positive tumors, which may be associated with enhanced tumor cell migration and invasiveness.

### 3.2. Immunohistochemistry (IHC)

Five proteins were selected for the validation of our findings by IHC staining of an HNSCC tissue micro array (TMA) containing 205 OPSCC samples with known p16 status (Table S2). While MCM2, 1amin B1 and NUP210 had shown a strongly enhanced expression in HPV-positive tumors as described above, two multifunctional proteins with reduced expression in HPV-positive tumors were further selected based on their clinical relevance and biological function. SLC3A2 (solute carrier family 3 member 2) comprises the heavy chain of the heterodimeric amino acid transporter CD98. Beyond or as a consequence of amino acid metabolism, SLC3A2/CD98 are involved in a wide range of functions relevant for cancer development and survival, such as mTOR pathway activation and autophagy [58], integrin signaling [59], immunity [60] and oxidative stress [58,61] and are negative prognostic factors in various cancers [62,63]. In HNSCC, SLC3A2/CD98 were described to mark tumor stem cell populations [64] and to confer a negative prognosis, which was completely restricted to HPV-positive tumors in one report [65] but primarily described for HPV-negative tumors in others [58,66,67,68]. In contrast to SLC3A2, there are no data regarding a possible prognostic role of leucine-rich PPR motif-containing protein (LRPPRC) in HNSCC. The protein is mostly localized at the mitochondria but also in the nucleus, at the nuclear membrane, ER and cytoskeleton and has been associated with various functions. Amongst others, LRPPRC is implicated in mitochondrial RNA metabolism acting as an RNA-chaparone for the mitochondrial transcriptome [69], is reported to be a restriction factor for autophagy, mitophagy and apoptosis [70] and was recently described to be a negative regulator of the mitochondrial antiviral signaling protein (MAVS) during viral infection [71]. Although the retinoic acid-inducible gene I protein (RIG1)/MAVS pathway normally responds to RNA viruses, it has also been described to play a role in the innate defense against HPV, although the molecular source of pathway stimulation is currently unknown [72]. An altered expression of LRPPRC has been reported in various tumors, with high levels often being associated with worse prognosis [70].

**Figure 6:**
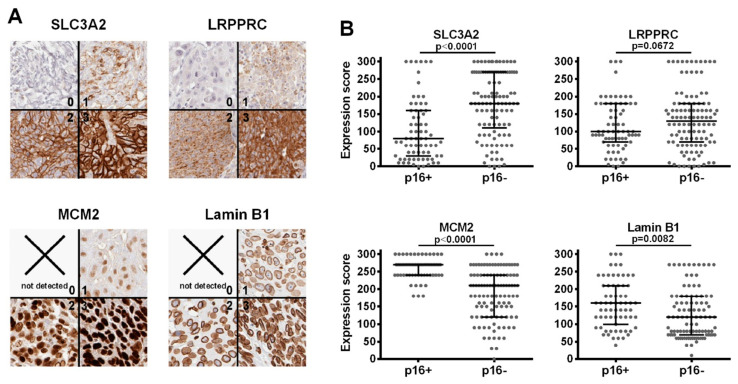
Validation of differential expression through IHC-analyses in an OPSCC tissue microarray. (**A**) Examples of staining intensities classified 0 (no), 1 (weak), 2 (moderate) and 3 (strong). (**B**) Semiquantitative expression scores (“maximum intensity” * “respective % of cells stained”) confirm significantly different expression of SLC3A2, MCM2 and lamin B1 dependent on p16-status (unpaired, one-sided *t*-test). Note that the semiquantitative scoring may underestimate expression differences, since factors of 1, 2 and 3 are cautious estimations when appraising the expression differences between weakly, moderately and strongly stained tumors. Depicted are median ± interquartile range.

With the exception of NUP210, suitable staining conditions could be established for all proteins (Figure 6A). Creating a semiquantitative expression score by multiplying the highest staining intensity observed (0 to 3) with the respective percentage of tumor cells stained, we could validate a significantly higher expression of MCM2 and lamin B1 and lower expression of SLC3A2 in the p16-positive samples and further observed a non-significant trend towards reduced expression for LRPPRC (*p* = 0.0678, one-sided *t*-test) (Figure 6B).

**Figure 7:**
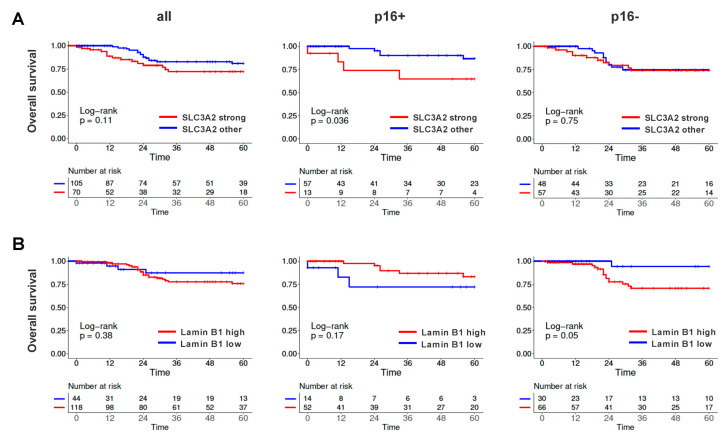
Patient survival in dependence of the SLC3A2 or LRPPRC expression status. (**A**) Overall survival in dependence of SLC3A2 and p16 status. OPSCC were categorized as showing strong (3) vs. all other (0,1,2) staining intensities. (**B**) Overall survival in dependence of lamin B1 and p16 status. OPSCC were categorized as showing a low (0,1) vs. high (2,3) staining intensity.

Regarding patient outcome, we observed significantly worse overall survival (OS) for the few patients whose p16-positive tumors demonstrated the highest (3) SLC3A2 staining intensity (*p* = 0.036), despite a considerable association of SLC3A2 expression with lower N-stage. No such difference was observed in patients with p16-negative tumors (Figure 7A, Figure S4B). A low lamin B1 staining intensity (0 and 1) conferred an excellent prognosis for patients with p16-negative tumors (*p* = 0.05), but rather trended towards worse survival in those with p16-positive ones (*p* = 0.17) (Figure 7B). Interestingly, these differences with regard to p16-status were not observed for recurrence-free survival (RFS). Here, higher SLC3A2 staining intensity trended towards worse survival independently of p16-status, resulting in significance for the whole OPSCC population. Lamin B1 staining intensity had no impact on RFS in both entities (Figures S4 and S5). The underlying mechanisms for these differences between OS and RFS require elucidation in future studies. No prognostic impact was observed for MCM2 or LRPPRC.

## 4. Discussion

Our study represents the third mass spectrometric comparison of HPV-positive and –negative OPSCC. Slebos et al. performed repeated measurements of pooled tissue samples from 10 HPV-positive vs. 10 HPV-negative frozen tumors [73]. A disadvantage of the use of pooled extracts is that inter-tumor heterogeneity is not assessed and outliers with high expression can have a large impact. To minimize the rate of false positive results, we integrated the inter-tumor heterogeneity in protein expression levels by performing individual measurements of each tumor specimen and by applying a random forest machine learning approach to identify proteins that can separate the two groups. When independent data sets are available, concordant results can be considered a strong indicator for real differences. Therefore, we also classified those proteins as unequally expressed whose intensities were significantly and, on average, at least two fold different in our and the comparable study by Sepiashvili et al., the largest mass spectrometric analysis of HPV-positive and –negative OPSCC to date [29]. In total, this yielded a number of 31 differentially expressed proteins. Of the 16 proteins identified solely through the random forest analysis, 10 could be confirmed by the Sepiashvili dataset when applying relaxed criteria (one sided unadjusted t-test with same direction of enhanced/reduced expression), while for five proteins (SLC3A2, CCT8, HEBP2, RhoA, histone H2B isoforms), a differential expression could not be confirmed and one protein (INPP1) had not been detected.

Within our dataset, two factors clearly speak in favor of the accuracy and robustness of our measurements: i. the detection of p16 in seven of eight HPV-positive but in none of the HPV-negative samples (Figure 2B) and ii. the tight association of the expression levels of proteins known to be co-regulated, namely the MCM-subunits as well as lamin B1 and LAP2 (Figure 4, Figure S3). Finally, we could confirm a significantly different expression of three out of four proteins tested by IHC analysis of a large independent cohort on a tissue micro array (Figure 6).

### 4.1. Implications for Tumor Biology

Regarding tumor cell biology, our results suggest differences between HPV-positive and HPV-negative OPSCC, especially in three functional groups: replication, nuclear architecture and cytoskeleton regulation. The expression of replication factors, especially the MCM-proteins, was congruently found to be upregulated in HPV-positive OPSCC in all three proteome studies as well as previous transcriptome analyses [29,73,74,75,76]. The non-tumorigenic normal life cycle of HPV necessitates the transfer of differentiating cells into the replicative S-phase to enable the productive replication of the viral genome [77]. The most important mechanism for this forced S-phase entry is the degradation of pRB and subsequent liberation of the transcription factors of the E2F-family, which govern the expression of many S-phase specific genes. For example, the expression of the MCM-proteins and LAP2, are at least partly governed through E2F-induced transcription [78,79]. Interestingly, the protein levels of LAP2 and lamin B1 are normally co-regulated together with pRB by the ubiquitin ligase RNF123 [44] and RBBP4/7 and LAP2 are direct binding partners of pRB [80,81]. An enhanced RBBP4 expression has been previously described in an HPV-unrelated experimental tumor model based on somatic inactivation of RB [82]. Therefore, the enhanced expression of these proteins in HPV-positive HNSCC may well be a direct or indirect consequence of the E7-mediated lack of pRB in these tumors, which confirms the findings from previous studies [29,73,74,75,76].

The most relevant clinical features of HPV-positive OPSCC are the enhanced sensitivity towards treatment, especially radiotherapy [8,13], and early formation of lymph node metastases. The latter requires a high migratory and invasive capacity, which may be reflected by the enhanced expression of various cytoskeletal regulators. In fact, high expression of gelsolin, CapG, Stathmin, CCT8 and Rho GTPases have all been described to promote tumor cell migration, invasion and formation of metastases in various tumor entities [83,84,85,86,87,88,89,90,91]. For gelsolin, a direct interaction with HPV-E7 has been demonstrated to exert anti-apoptotic and pro-survival effects [92] and to be critical for cell movement and invasiveness in cervix carcinoma [93]. In contrast, the cytoskeletal regulator cortactin, which recruits the ARP2/3 complex to existing actin filaments [94], was among the few proteins expressed at a reduced level in HPV-positive OPSCC (see Figure S1). This may raise the possibility that the enhanced ARP3 level may to some extent represent a compensatory mechanism.

Regarding the enhanced radiation sensitivity of HPV-positive OPSCC, we had previously shown that HPV-positive HNSCC cells are characterized by a defect in the repair of radiation-induced DNA double-strand breaks [17], which was confirmed in further reports [13,15]. Some of the differences observed in our analysis may potentially have a profound impact on chromatin organization, which may also affect DNA repair after irradiation. For example, the nuclear lamina and NUMA1 are well described to participate in chromatin organization [37,38,43]. In this regard, we observed a negative prognostic impact of high lamin B1 expression in HPV-negative but not HPV-positive OPSCC in the TMA analyses (Figure 7). While this is an interesting finding, its understanding clearly requires further investigations. RBBP4/7 are not only part of the chromatin assembly factor complex (CAF-1), which reinstalls chromatin after replication and repair but represent the histone binding component of various chromatin remodeling complexes: the nucleosome remodeling and histone deacetylase (NuRD) complex, the core histone deacetylase (HDAC) complex, the nucleosome remodeling factor (NURF) complex and the PRC2/EED-EZH2 complex, all of which regulate, mostly repress, transcription and have been implicated in oncogenesis [95,96,97]. It is obvious that altered expression of global chromatin organizing factors may have various potential implications, but to clarify their role in the treatment sensitivity of OPSCC requires future functional studies. Potentially influencing DNA repair and radiation sensitivity in a direct manner, Numa1 has been described to promote homologous recombination repair [40] but to inhibit the non-homologous endjoining promoting factor 53BP1 by restricting its diffusion in the nucleoplasm [39]. RBBP4 on the one hand and SLC3A2 and cortactin on the other have been explicitly described as radiosensitivity or radioresistance factors, respectively [58,65,66,98,99,100]. Intriguingly, SLC3A2 was shown to mediate radioresistance and to be a negative prognostic factor in HPV-negative HNSCC cells and tumors in some recent reports [58,66,68], whereas our overall survival data rather support the finding of a generally reduced expression and of a negative prognostic role more evident in HPV-positive OPSCC as reported by Rietbergen et al. [65] (Figure 7). Whether and by which means SLC3A2 may actively contribute to the inferior survival and whether it may also serve as a predictive marker, e.g., for the exclusion of patients with HPV-positive tumors from de-intensified regimes, will need to be addressed in future studies.

Another interesting finding of our analysis is the enhanced expression of ubiquitin and of S-phase kinase-associated protein 1 (SKP1), an essential component of the SCF (SKP1-CUL1-F-box protein) E3 ubiquitin ligase complex. Target specificity of the SCF complex is mediated by the specific member of the F-box protein family loaded. Ubiquitination mediates degradation via the 26S-proteasome but also the activity of a large number of proteins. Amongst other functions, SCF-complexes are critically involved in the regulation of the DNA damage response and double-strand break repair by mediating the degradation or triggering the activity of central factors, such as NBS1, Exo1, claspin, XRCC4 or CDC25A [101,102,103,104]. Linking the 26S proteasome/ubiquitin system to radiation sensitivity, a decreased expression of components of this pathway was congruently associated with a more radioresistant phenotype in breast, laryngeal, lung, esophageal and rectal cancer and/or cancer cell lines [105,106,107,108,109,110], which is in line with the observation of enhanced expression of ubiquitin and SKP1 in the radiosensitive HPV-positive OPSCC population. Finally, recent data suggest critical roles of nuclear actin and the actin regulating ARP2/3 complex in DNA repair, e.g., by mediating the movement of double-strand breaks out of densely packed heterochromatic areas [111,112] but to date, no connections to the cellular radiation sensitivity have been drawn.

### 4.2. Limitations of Our Study

Our exploratory mass spectrometric study has several limitations, such as a limited number of tumors or the inclusion of tumor-associated stroma cells in the macrodissected tissue samples, which will to some extent, dilute the tumor-cell-specific signals. In addition, the detected peptides were not always sufficient to distinguish between isoforms or homologous proteins. For example, the three Rho proteins RhoA, RhoB and RhoC, which share 85% sequence identity [113], were identified by two peptides in our mass spectrometric analysis (Table S1). Since these peptides are identical in the three Rho proteins, it cannot be inferred whether one, two, or all three Rho proteins were present in our samples and whether there was any difference within the Rho protein composition. The use of whole tissue extracts clearly favors the detection of proteins with high abundance or highest desolvation and ionization efficiency. Despite these limitations, we were able to detect meaningful differences between the two OPSCC entities. The tumorigenesis of HPV-positive cancers is to a large extent mediated by the multifunctional viral oncoproteins E6 and E7. These tumors are, therefore, expected to represent a relatively homogeneous group, as compared to carcinogen-induced tumors largely driven by randomly occurring mutations, which may help to detect differences between both entities. A puzzling finding of our study is the by far higher number of proteins with higher expression in HPV-positive tumors (27 proteins) compared to those with a higher expression in HPV-negative (4 proteins). While for us unexpected, this observation is highly concordant with the results from the Sepiashvili study [29]. A given higher homogeneity of HPV-positive tumors may again be a contributing factor as different HPV-negative tumors may less uniformly overexpress the same proteins and those factors potentially downregulated in HPV-positive tumors may not have been detected in enough samples. In general, data from open, exploratory approaches like ours can provide novel insights but should be considered as hypothesis generating and mostly require independent validation and more detailed and functional follow-up analyses.

## 5. Conclusions

In summary, our mass spectrometric comparison of HPV-positive and negative OPSCC tumors has identified protein level differences in replication, nuclear architecture and cytoskeletal regulation. We further identified a number of potential HPV surrogate markers and confirmed, respectively identified the negative prognostic value of strong expression of SLC3A2 and lamin B1 in HPV-positive and HPV-negative OPSCC, respectively. Finally, our data suggest several candidate proteins, which, based on altered expression in HPV-positive tumors, may contribute to the enhanced treatment sensitivity.

## Figures and Tables

**Table 1:** Clinicopathological characteristics of OPSCC used in this study. Statistical analyses were performed using a two-sided Mann–Whitney test (age) or Fisher’s exact test (all others) (R, version 3.6.1). ECS = extracapsular spread, ed. = edition.

Cohort	p16-Positive	p16-Negative
Number of patients	8	9
Age, median (range) (*p* = 0.6993)	64.9 (59–76)	66.8 (53–83)
Sex (*p* = 1)		
Male	6	7
Female	2	2
pT classification (*p* = 0.8756)		
T1	4	6
T2	2	2
T3	1	1
T4	1	0
pN classification (*p* = 0.1316)		
N0	0	4
N1	3	1
N2	4	4
N3	1	0
TNM stage (7th ed.) (*p* = 0.2467)		
I	0	2
II	0	2
III	3	1
IV	5	4
ECS (*p* = 1)		
Pos	3	3
Neg	5	6
smoking (*p* = 0.2941)		
Yes	5	8
No	3	1

**Table 2:** Proteins differentially expressed in HPV-positive and HPV-negative HNSCC. Proteins 1–24 were identified as unequally expressed through the random forest analysis, proteins 25–31 were additionally identified through significance (unadjusted) and fold change (difference ≥ 2 fold, corresponding to Log2FC values ≥1 or ≤−1) congruently in this and a comparable previous study [29]. Bold numbers indicate that the respective thresholds were met.

No	Gene (Protein) Names	Boruta Score	*p*-Value	Log2FC (HPV+/−)	*p*-Value (Sepiashvili et al.) [29]	Log2FC (HPV+/−) (Sepiashvili et al.)
1	*MCM2*	**200**	**0.000235**	**2.8231**	**0.000573**	**1.1887**
2	*NUP210*	**200**	**0.000261**	**1.3284**	**0.000764**	**1.5418**
3	*LMNB1* (Lamin B1)	**200**	**0.000651**	0.8628	**0.002252**	0.5517
4	*TOP2B*	**200**	**0.004052**	**1.3653**	**0.007912**	0.6057
5	*CDKN2A* (p16)	**200**	**0.004598**	**1.3982**	**0.000002**	**3.3464**
6	*RHOA*; *RHOC*; *RHOB*	**200**	**0.012392**	0.8633	0.6526 (*RHOA*)	0.0336 (*RHOA*)
7	*STMN1*; *STMN2* (Stathmin; Stathmin-2)	**196**	**0.001886**	**1.0479**	0.0544 (*STMN1*); **0.0050** (*STMN2*)	0.3838 (*STMN1*) **1.1807** (*STMN2*)
8	*ACTR3* (ARP3)	**196**	**0.005275**	0.4038	**0.042775**	0.3757
9	*UBA52*; *RPS27A*; *UBB*; *UBC*	**132**	**0.001915**	0.4820	0.0601 (*UBA52*); 0.0765 (*RPS27A*)	0.3174 (*UBA52*); 0.3108 (*RPS27A*)
10	*SKP1*	**72**	**0.001183**	**2.0275**	**0.015037**	**2.1586**
11	*HEBP2*	**46**	0.056098	0.8142	0.186598	0.4573
12	*HLA-DRB1*; *HLA-DRB5*	**40**	**0.002618**	**2.6530**	**0.006311**	0.8931
13	*CAPG*	**34**	**0.002681**	0.8428	**0.000616**	0.7927
14	*HIST1H4A*	**34**	**0.011118**	0.5163	0.076559	0.2950
15	*HIST2H2BE*; *HIST1H2BB*; *HIST1H2BO*; *HIST1H2BJ*; *HIST3H2BB* (Histone H2B, multiple types)	**24**	**0.010950**	0.7303	0.1806 (*HIST1H2BB*); 0.1988 (*HIST1H2B*J); 0.1244 (*HIST3H2BB*)	0.2649 (*HIST1H2BB*); 0.2754 (*HIST1H2B*J); 0.3035 (*HIST3H2BB*)
16	*INPP1*	**20**	**0.041938**	**1.2062**	n. d.	n. d.
17	*MCM5*	**18**	**0.002374**	**1.5963**	**0.005306**	**1.3743**
18	*LRPPRC*	**18**	0.085066	**−1.1076**	0.060111	−0.5621
19	*MCM6*	**16**	**0.007168**	**1.5859**	**0.000426**	**1.2566**
20	*CCT8*	**14**	**0.022356**	0.4512	0.463460	0.1661
21	*TMPO* (LAP2)	**12**	**0.005542**	**1.0096**	**0.016226**	**1.0534**
22	*RBBP4;RBBP7*	**10**	**0.003404**	**1.1455**	0.0707 (RBBP4) 0.9507 (RBBP7)	−0.4031 (RBBP4) −0.0165 (RBBP7)
23	*SLC3A2*	**10**	**0.006046**	**−1.5631**	0.317565	−0.4671
24	*GSN* (Gelsolin)	**10**	**0.027809**	0.6165	**0.029810**	0.3432
25	*CTTN* (Cortactin)	1	**0.015119**	**−1.2674**	**0.000396**	**−1.2752**
26	*MCM3*	0	**0.014093**	**1.5990**	**0.000023**	**1.3652**
27	*NUMA1*	0	**0.016250**	**1.5247**	**0.000340**	**1.1813**
28	*MCM7*	0	**0.028008**	**1.2418**	**0.000820**	**1.0757**
29	*AKR1B10*	0	**0.035758**	**−2.6277**	**0.042775**	**−1.0294**
30	*ICAM1*	0	**0.037439**	**1.2982**	**0.000168**	**3.5303**
31	*PTPRC*	0	**0.042037**	**1.0681**	**0.012870**	**1.0362**

## Supplementary Materials

The following are available online at https://www.mdpi.com/2072-6694/12/6/1531/s1, Figure S1: Proteins identified to be expressed at a higher level in HPV-negative OPSCC as assessed by LC-MS/MS intensity values, Figure S2: Protein-protein interaction network. The network was created with the STRING tool. Line colors indicate the type of interaction (grey = unspecified), arrowheads indicate a positive, negative or unspecified effect. Replication factors: MCM2, MCM3, MCM5, MCM6, MCM7, RBBP4; Nuclear architecture: Lamin B1, LAP2, NUP210, Numa1; Cytoskeleton regulators: APR3 (ACTR3), Gelsolin (GSN), CAPG, Stathmin (STMN), RhoA, Cortactin (CTTN), Numa1, CCT8, Figure S3: Linear regression analyses demonstrate tight associations of the expression levels of the depicted MCM proteins as assessed by LC-MS/MS intensity values, Figure S4: Recurrence free survival in dependence of the SLC3A2 expression status and correlation with clinicopathological characteristics, Figure S5: Patient survival in dependence of the lamin B1 expression status, Table S1: Complete list of proteins identified in the mass spectrometric analysis, including label-free quantification (LFQ) values, numbers of unique peptides (UP), Boruta score, t test, and observations from Sepiashvili et al. for each protein, Table S2: Clinicopathological characteristics of tissue microarray OPSCC samples.
