# Supplementary material for: Mass Spectrometric Comparison of HPV-Positive and HPV-Negative Oropharyngeal Cancer
Source: Cancers (Basel). 2020 Jun 11;12(6):1531. doi: 10.3390/cancers12061531 (PMC7352546; doi:10.3390/cancers12061531)
Supplement: Supplementary file 1 [file cancers-12-01531-s001.zip › cancers-829216-suppl-XML/cancers-829216-Figures S1-S5 and Table S2.pdf]

Supplementary Materials: Mass Spectrometric Comparison of HPV-Positive and HPV-Negative Oropharyngeal Cancer

Marcus Wurlitzer, Nikolaus Möckelmann, Malte Kriegs, Maren Vens, Maryam Omidi, Konstantin Hoffer, Clara von Bargen, Christina Möller-Koop, Melanie Witt, Conrad Droste, Agnes Oetting, Hannes Petersen, Chia-Jung Busch, Adrian Münscher, Hartmut Schlüter, Till Sebastian Clauditz and Thorsten Rieckmann

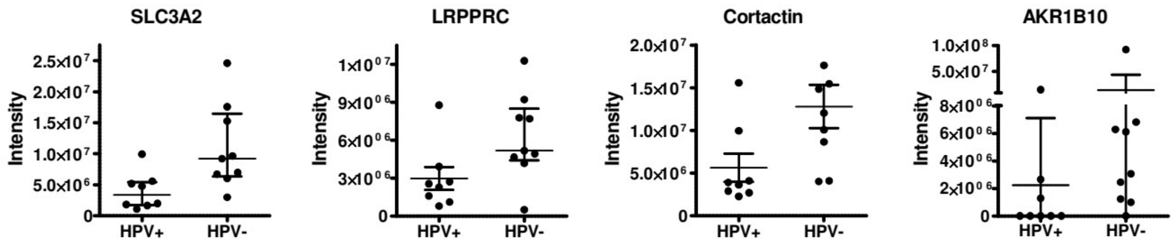

Figure S1. Proteins identified to be expressed at a higher level in HPV-negative OPSCC as assessed by LC-MS/MS intensity values.

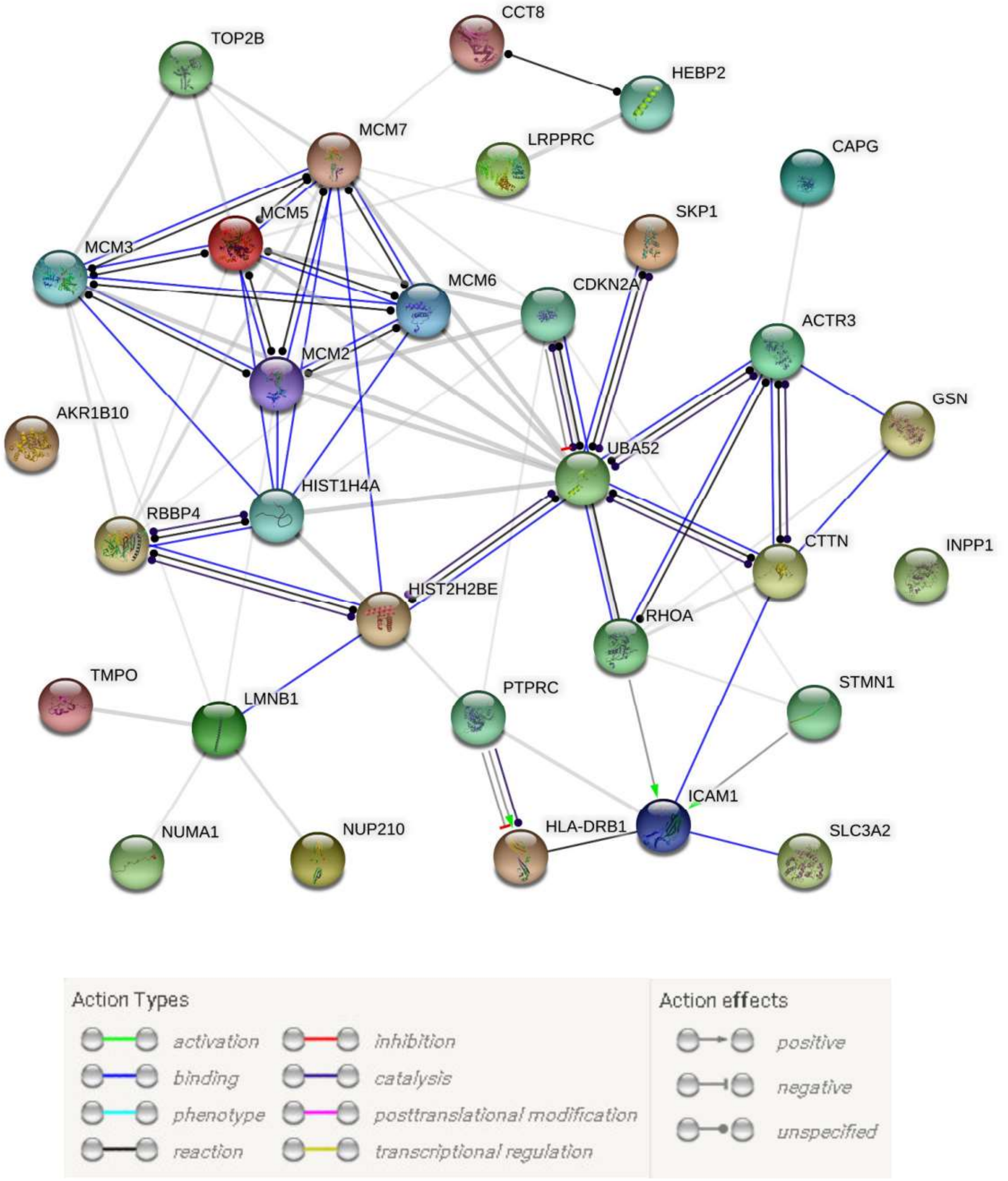

Figure S2. Protein-protein interaction network. The network was created with the STRING tool. Line colors indicate the type of interaction (grey = unspecified), arrowheads indicate a positive, negative or unspecified effect. Replication factors: MCM2, MCM3, MCM5, MCM6, MCM7, RBBP4; Nuclear architecture: Lamin B1, LAP2, NUP210, Numa1; Cytoskeleton regulators: APR3 (ACTR3), Gelsolin (GSN), CAPG, Stathmin (STMN), RhoA, Cortactin (CTTN), Numa1, CCT8.

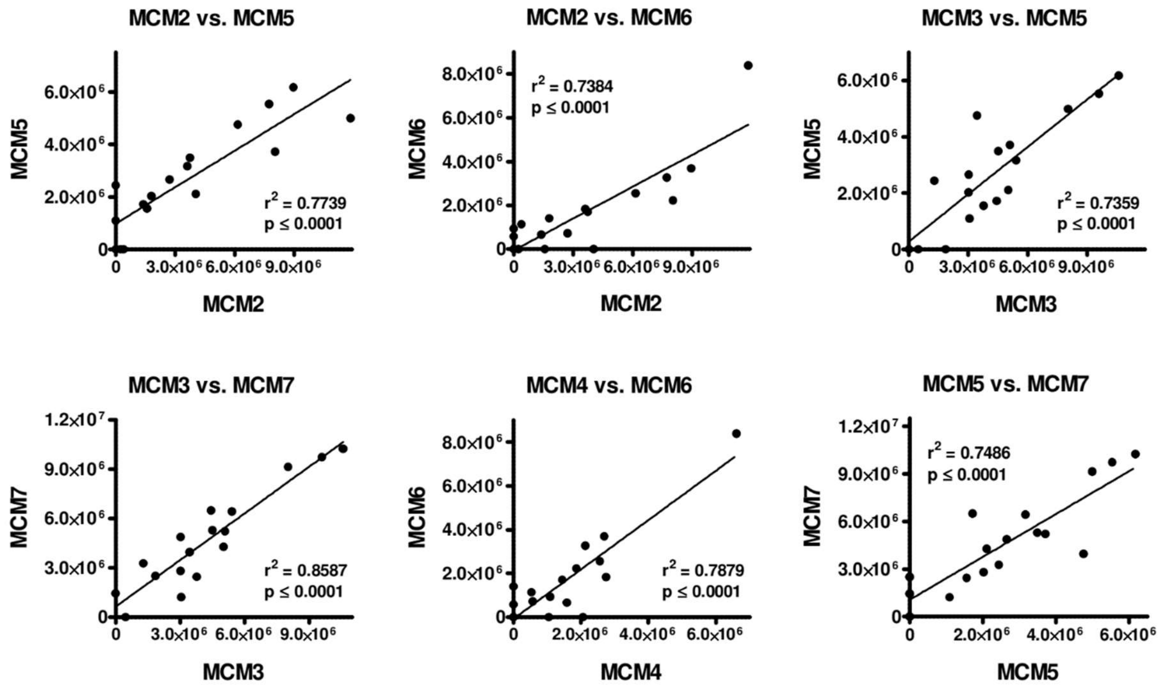

Figure S3. Linear regression analyses demonstrate tight associations of the expression levels of the depicted MCM proteins as assessed by LC-MS/MS intensity values.

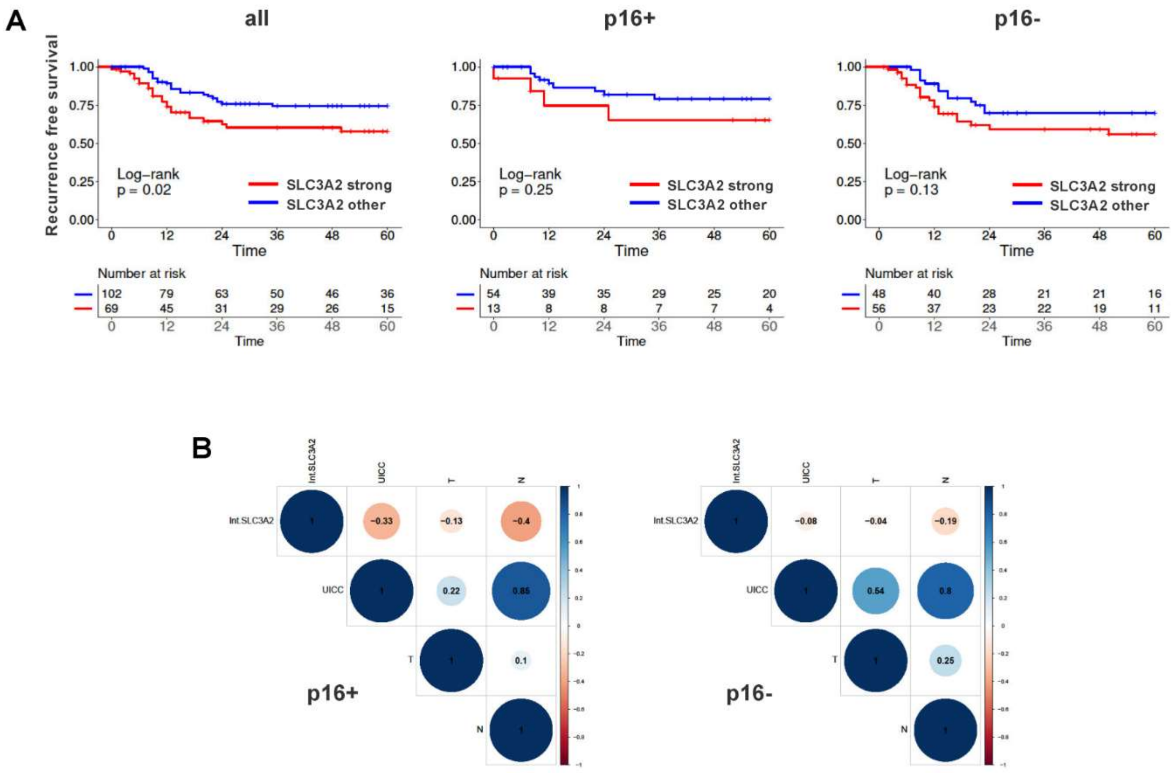

Figure S4. Recurrence free survival in dependence of the SLC3A2 expression status and correlation with clinicopathological characteristics. (**A**) RFS. Patients were categorized as showing strongest (3) vs. all other staining intensities (0,1,2). (**B**) Correlation analyses of SLC3A2 staining intensity with UICC (7th edition), T and N-stage in dependence of the p16 status. Note that the negative prognostic impact of high SLC3A2 in p16-positive OPSCC exists despite a negative association with UICC and

N-stage. This is in line with the finding that N-stage has little prognostic relevance in p16-positive OPSCC (and has therefore been excluded in the 8th edition of UICC-staging).

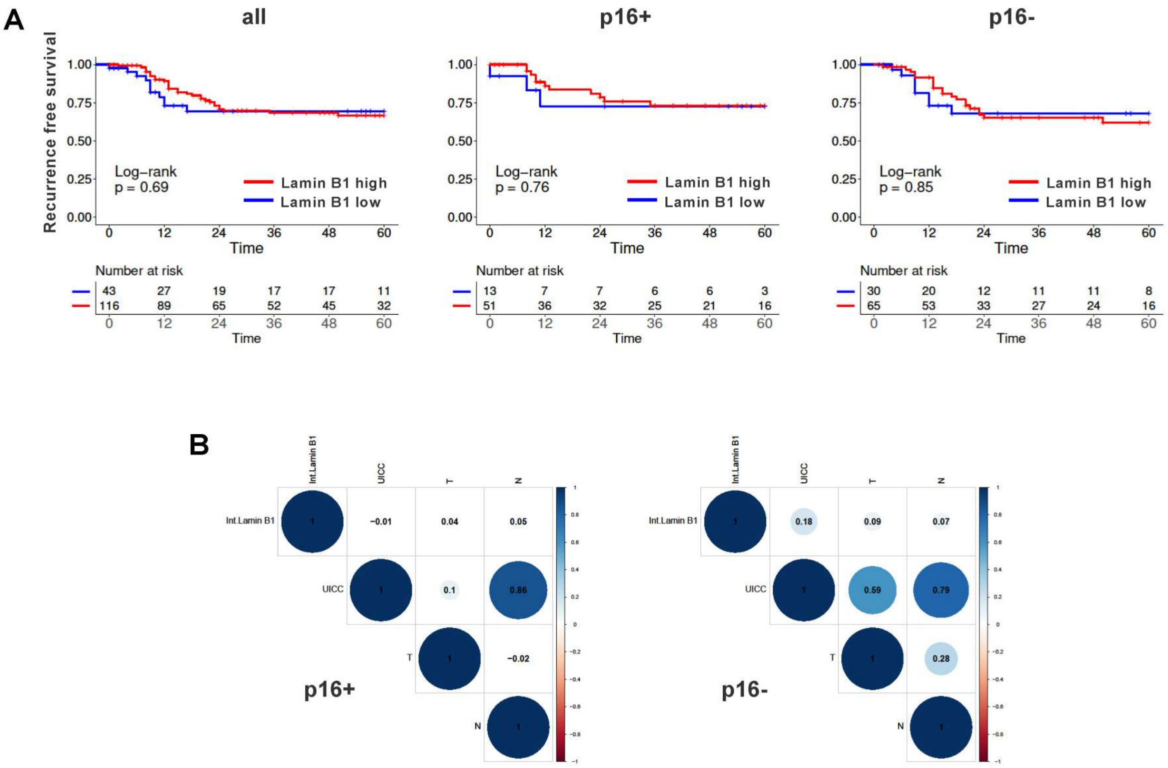

Figure S5. Patient survival in dependence of the lamin B1 expression status. (**A**) Recurrence free survival in dependence of lamin B1 and p16 status. Patients were categorized as showing low (0,1) vs. high (2,3) staining intensities. (**B**) Correlation analyses of lamin B1 staining intensity with UICC (7th edition), T and N-stage (top row) in dependence of the p16 status does not suggest considerable associations.

Table S2. Clinicopathological characteristics of tissue microarray OPSCC samples.

Cohort	p16-Positive	p16-Negative
Patients, number	78	127
Age, median (range)	59.5 (32-82)	60 (43-85)
Sex, number (%)		
male	63 (80.8)	97 (76.4)
female	15 (19.2)	30 (23.6)
pT classification, number (%)		
T1	17 (21.8)	37 (29.1)
T2	29 (37.2)	37 (29.1)
T3	23 (29.5)	21 (16.5)
T4	9 (11.5)	32 (25.2)
pN classification, number (%)		
N0	15 (19.2)	47 (37)
N1	12 (15.4)	17 (13.4)
N2	43 (55.1)	55 (43.3)
N3	8 (10.3)	8 (6.3)
TNM stage (7th edition), number (%)		
I	3 (3.9)	17 (13.4)
II	6 (7.7)	17 (13.4)
III	19 (24.4)	23 (18.1)
IV	50 (64.1)	70 (55.1)
Therapy, number (%)		
surgery	14 (17.9)	44 (34.6)
surgery + (chemo)radiation	47 (60.3)	49 (38.6)
chemoradiation	8 (10.3)	21 (16.5)
radiotherapy	2 (2.6)	5 (3.9)
other	1 (1,3)	1 (0.8)
n.a.	6 (7.7)	7 (5.5)
Interpretable staining, number (%)		
SL3CA2	71 (91)	108 (85)
LRPPRC	72 (92.3)	109 (85.8)
MCM2	68 (87.2)	108 (85)
lamin B1	67 (85.9)	98 (77.2)

© 2020 by the authors. Licensee MDPI, Basel, Switzerland. This article is an open access article distributed under the terms and conditions of the Creative Commons Attribution (CC BY) license (http://creativecommons.org/licenses/by/4.0/).
